# The Surgical Treatment of Cholelithiasis*Delivered before Richmond Academy of Medicine and Surgery, September 27, 1895.

**Published:** 1895-11

**Authors:** Hugh M. Taylor

**Affiliations:** Richmond, Va., Professor of Practice of Surgery in University College of Medicine, Surgeon to Virginia Hospital, etc.


### THE SURGICAL TREATMENT OF CHOLELITHIASIS.*

By HUGH M. TAYLOR, M. D., RICHMOND, VA.,

Professor of Practice of Surgery in University College of Medicine, Surgeon to Virginia Hospital, etc.

The surgery of the gall tract is perhaps claiming a professional interest second only to that accorded to appendicitis. The one morbid condition is as essentially surgical as the other, and both are equally responsible for ill-health and death. The evolution of the subject of appendicitis received an impetus earlier. It followed close upon the concentration of thought on the pelvic phlegmons, but in view of its importance gall-tract surgery is receiving its merited share of attention. Few of us can review our professional work and not be conscious of having treated operable cases of cholelithiasis and its consequences unrecognized as such. The evolution of the subject has been rapid. Within the past ten or twelve years all the marked advance in its study has been made, and, while already yet in its infancy, the field of operative procedure has been immensely widened and many of its morbid conditions have been brought within the scope of legitimate conservative surgery, with untold benefit to mankind. Viewed in the light of recent knowledge, we appreciate the fact that few of us have failed to treat as gastralgia, indigestion, diaphragmatic pleurisy, enlargement of the liver, malarial fever, bilious fever, etc., cases which should have been diagnosed and treated as cholelithiasis and its consequences. In my own early professional work I can recall at least a half-dozen clearly operable cases which were allowed to go from bad to worse and die without operative aid. Enough has been ascertained to prove that the so-called medical treatment offers but a small chance of benefiting the condition of impacted gall-stone or stones, and certainly no possible chance of curing a cholangitis or empyema or cystitis of the gall-bladder. Experience shows that the solution of gall-stones by medication is a myth, and that whenever they have attained to any size, or are present in considerable numbers, and are producing symptoms, they can only be

*Delivered before Richmond Academy of Medicine and Surgery, September 27, 1895.

rationally treated by operation. Moreover, it is true that a laparotomy *per se* for the relief of such conditions is attended with less shock and danger than is a laparotomy for other intraperitoneal troubles, as there will usually be very much less evisceration. This much in connection with the subject of cholelithiasis is practically settled. The unsettled problem is to classify the various morbid conditions; to differentiate clinically between them prior to an exploratory incision, and to apply to their relief the most rational available surgical procedure. In this we have a field for experimental pioneer work, the cultivation of which offers rich reward.

What are the morbid conditions calling for surgical interference? By almost unanimity of opinion, and specifically, according to an enumeration of Prof. W. Mayo Robson, operative interference is indicated and the earlier the better:

(*a*) "In cases of repeated attacks of biliary colic, apparently due to gall-stones, which, not yielding to medical treatment, are wearing out the patient's strength.

(*b*) In perforation from ulceration.

(*c*) When there is suppuration in the neighborhood of the gall-bladder set up by gall-stones.

(*d*) In empyema of the gall-bladder, which is usually accompanied by peritonitis.

(*e*) In dropsy of the gall-bladder.

(*f*) In obstructive jaundice, when there is reason to think that the common duct is occluded by gall-stones."

The study of the subject of cholelithiasis and its consequences will be facilitated if we keep in mind the fact that the gall-tract is a drain-tract, through which the bile from the liver and the mucus from the gall-bladder must pass unobstructed to ensure good health. Obstruction means the damming back of the outflowing products, dilatation of the ducts or gall-bladder, irritation of the tracts, suppuration in the tracts, extension of the inflammation to the peritoneal investment of the tracts, adhesions and matting around the inflamed tube, and a pathological condition analogous to inflammation in the Fallopian tubes and vermiform appendix. While a catarrhal cholangitis, the inflammation limited to the mucous lining of the tubes, may be possible, usually all the coats (the peritoneal included) are involved.

We should also keep well in mind that while an obstruction in the cystic duct dams back the secretions of the gall-bladder only, an obstruction in the common duct dams back the bile from the liver as well as the mucus from the gall-bladder. Obstruction of the cystic induces dropsy of the gall-bladder, empyema, chronic cystitis, inflammation of its coats, including the peritoneal investment, perforation or gangrene, and the local and constitutional symptoms incident to such morbid conditions.

Obstruction in the common duct induces inflammation of the duct, extension of that inflammation to the peritoneal investment, local peritonitis, adhesions, and, perhaps, ulceration and perforation, dilatation of the duct, decomposition of the retained products within the duct, and systemic poison as a consequence. From the irritation alone reflex disturbances are not infrequent, while the chills, fever, sweats, etc., make a septic cholangitis, simulating so closely malarial, bilious, and other febrile manifestations. There is no jaundice in cystic duct obstruction. Varying jaundice in common duct obstruction from stone. Persistent, unvarying jaundice in obstruction from neoplasms, benign or malignant, pressing on the tube, is the rule. Obstruction of the common duct, from whatever cause, if persistent enough, dams back the bile, induces cholæmia and its consequences; not the least serious of which are its effects upon the blood.

We should remember that stones in the gall-bladder do not necessarily give rise to trouble. It is estimated that ten per cent. of adult males, twenty-five per cent. of adult females, and thirty-six per cent. of the insane have gall-stones, and only from one to two per cent. have symptoms of the same. While this estimate of cases giving trouble is too small, it helps to sustain the well-known fact that many gall-bladders are full of stones which are doing no harm, but, then, again, they may give trouble by irritation, accumulation, infection, and inflammatory changes, often of an intense type. Each attack of inflammation of the gall-bladder due to stones therein, and with no obstruction, causes more or less thickening of its walls and subsequent contraction of the bladder until finally nothing remains but a bound down tube. Stones in the cystic duct, or stenosis, induce changes in the gall-bladder and duct, septic, ulcerative, or gangrenous, and colic, frequent and exhausting. Stones in the common duct induce urgent symptoms and call for prompt relief.

The dangers of cholelithiasis and its consequences as grouped are:

(*a*) "That repeated attacks may, and not infrequently do, exhaust the patient. Cases are on record in which the extreme vomiting and prolonged suffering of one attack has terminated fatally.

(*b*) Fatal cholæmia, with its strong hemorrhagic tendency, both post and ante operative.

(*c*) Distension of the gall-bladder until it enlarges sufficiently, in some cases, to reach to the pelvis, pressure effects incident thereto, and local and general effects from the decomposition of its retained products—*i. e.*, empyema and cystitis."

What operation shall be done, and how it shall be done, depends upon the indications to be met by operative interference. It goes without saying that not every case of cholelithiasis demands an operation. Gall-stones in great numbers are found in the gall-bladder without having induced symptoms of their presence, the patient continuing in good health. Under such circumstances there is no indication and no need for surgical aid. Only when symptoms incident to gall-stones are marked and continuous are we justified in interfering surgically. This justification will not, however, be wanting sooner or later if obstruction from stone, stricture, or morbid growth exists. Under such circumstances interference is imperative. The sooner the better, and this conclusion is fully sustained by the good results which are daily accumulating.

What are the operations applicable to the relief of gall-tract troubles? Prominent among the operative procedures which are now and have been in vogue for the past ten years are cholecystotomy, cholecystostomy, cholecystectomy, cholecystenterostomy, and choledochotomy. Of these the operations upon which most interest is being centered, the comparative value of which is, to some extent, a matter of dispute, are choledochotomy and cholecystenterostomy. Cholecystenterostomy was an advance of no small proportions over cholecystostomy. The technique of this operation, as now performed, is so simple, where the gall-bladder is not too much contracted; its immediate effects are so strikingly good; its execution attended with so little danger, that it was heralded as almost an ideal operation. Where the obstruction is in the cystic duct, when the damage is due to retention of the secretions of the gall-bladder, drainage into the duodenum is a great improvement over drainage through an incision in the abdominal parietes. When the obstruction is in the common duct, by cholecystenterostomy the current of bile is turned through the cystic duct and gall-bladder into the duodenum and its usefulness to the animal economy is not lost, as is the case where it escapes by means of a cholecystostomy. By it drainage of the suppurating gall-tract is secured and systemic poison—*i. e.*, cholæmia and septicæmia are prevented. Its ease of execution, its minimum death-rate, and its immediate effects for good mark it as an advance of large proportions. Nature pointed the surgeon to this way of draining the suppurating area around the gall-tract by not infrequently forming adhesions between the gall-bladder and duodenum and emptying the suppurating cavity into the intestine. For years various operators have essayed to imitate nature by trying to establish a gall-bladder and duodenal anastomosis by sutures; but not until the advent of that ingenious product of American invention—the Murphy Button—was the technique of cholecystenterostomy so simplified and so completely shorn of danger as to make it safe almost in the hands of the novice.

Dr. Murphy enumerates the indications for cholecystenterostomy as follows:

I. "In all cases where it is desirable to drain the gall-bladder.

II. In all cases of perforation into the abdominal cavity where the duct must be obliterated by the reparative process.

III. In all cases of cholelithiasis where obstruction of duct is present, or where the reflex disturbances of digestion are marked.

IV. In all cases of cholecystitis, either with or without gall-stones.

V. In all profusely discharging biliary fistulæ, either following operations or as a sequelæ of pathological changes in gall-tract."

It will be seen that Dr. Murphy finds in cholecystenterostomy, by means of his anastomosis button, a means for the relief of a large portion of the morbid conditions incident to cholelithiasis. His contraindications for its use are mainly a too much contracted gall-bladder to get the button in, where the adhesions are so extensive that we cannot get the duodenum up to the gall-bladder without risk of kinking it and inducing intestinal obstruction. Dr. Murphy himself and many operators, especially in this country, have furnished us with ample proof of the usefulness of this operation, which makes a new short route from the gall-tract to the intestinal canal. But a continued evolution of the subject of the surgical treatment of gall-tract diseases demonstrates to the satisfaction of many, whose opinions merit our regard, that cholecystenterostomy is not an ideal operation. It has a limited scope of application, and is only indicated for irremediable stenosis of the duct or where the impacted stone or obstruction, of whatever nature, cannot be removed. The ideal operation contemplates restoring the gall-tract to its natural state, re-establishing it as a drainage tract. This is accomplished by removing the obstruction —the cause of the morbid changes—which, in a majority of cases, is an impacted stone. The operation of choledochotomy—*i. e.*, incising the duct, removing the stone, immediate suturing of the duct, and drainage, as a precaution—is the ideal operation.

For a time surgeons hesitated to incise and suture the duct for fear of leakage of bile. Experience shows it to be safe and curative in the full sense of the word. Curative, in that, not only is drainage secured, but the cause of the morbid condition—the stone —is removed. As long as the stone remains in the duct it is an irritant, and inflammation, catarrhal or septic, and reflex gastric disturbances will continue. Cholecystenterostomy does not remove the stone impacted in the duct, and this is the weak point which limits its application. Choledochotomy is safe. The mortality of incising and suturing the duct is less than eighteen per cent.

It is true that it is not always an easy matter to find the duct and stone, and sometimes it is impossible either to locate the stone or remove it after it is located, or to bring the bound down duct into position to suture it; but an improved technique is rendering this a less formidable objection to choledochotomy. Dr. Elliott, of Boston, finds great advantage in placing the patient in a reversed Trendelenburg position, and also urges that the sutures to close the incision in the duct be passed before the exposed stone is removed. Some authors claim that a cholecystostomy should be done in connection with incision and suture of the duct, if we have an existing empyema of the gall-bladder or suppurating cholangitis, for the better drainage of the suppurating tract; but, others contend, if we remove the obstruction in the tubes, that sufficient and curative drainage will go on through the natural tract without additional aid. While many are better satisfied to drain with gauze the area of the sutured duct for a short time, still others, however, do not fear the leakage of bile and do not hesitate to close at once the abdomen.

An objection urged against cholecystenterostomy is the danger of infection of the gall-tract by the *bacillus coli communis* and infection of the liver; but, admitting this possible danger incident to establishing a short route for infection from the duodenum to the liver, it is of small consequence compared to the good resulting from free drainage in cases of empyema and cystitis of the gall-bladder from stenosis of cystic duct, or in cases of cholangitis and cholæmia from common duct obstruction which cannot be removed.

Choledochotomy and cholecystotomy are, unquestionably, ideal operations where they can be done. Cholecystenterostomy, with Murphy button, while it only relieves the consequences and does not remove the cause, has saved and will continue to save lives, especially by tiding over desperate cases too feeble from sepsis and cholæmia to stand a prolonged operation.

In many of its details the technique of the operation of choledochotomy is still imperfect, but, in spite of this, it is an ideal operation in its conception, and a mortality of less than eighteen per cent. is wonderfully encouraging as to results. In this as in other fields of abdominal surgery the point should be urged that fatalities are not due to the operation, but to the want of its early execution. While it is often impossible to make the diagnosis of cholelithiasis and its consequences, or utterly impossible, in many instances, to differentiate one morbid condition from the other, it is fair to assume that choledochotomy will be more than ever an ideal operation when we can diagnose gall-tract diseases before long existing cholangitis and local peritonitis have bound down the duct, matted the parts in its neighborhood, and poisoned the system by septic or cholæmic infection.

